# Integrating core muscle morphology into a predictive model for residual back pain after vertebral augmentation

**DOI:** 10.3389/fsurg.2026.1799571

**Published:** 2026-04-08

**Authors:** Mingyang Huang, Genzhong Xu, Ming Luo

**Affiliations:** 1 Department of Spine Surgery and Musculoskeletal Tumor, Zhongnan Hospital of Wuhan University, Wuhan, China; 2 Department of Emergency Surgery, The First Affiliated Hospital of Henan University of Chinese Medicine, Zhengzhou, China

**Keywords:** gluteal muscles, nomogram, osteoporotic vertebral compression fracture, percutaneous vertebral augmentation, residual back pain

## Abstract

**Background:**

Residual back pain (RBP) after percutaneous vertebral augmentation (PVA) for osteoporotic vertebral compression fractures (OVCF) remains a significant clinical challenge. Traditional prediction models focus primarily on bone mineral density and procedural factors. This study aimed to develop and validate a novel nomogram that incorporates the morphology of core muscles, notably the gluteal muscles, for personalized RBP risk stratification.

**Methods:**

In this retrospective study, clinical data from 428 OVCF patients who underwent PVA at two centers were analyzed. Patients were randomly divided into training and validation cohorts (3:1 ratio). Variables included demographics, fracture characteristics, procedural details, and computed tomography-based measurements of the relative cross-sectional area (rCSA) of paravertebral (multifidus, erector spinae, psoas) and pelvic [gluteus maximus (Gmax), gluteus medius (Gmed)] muscles. Least absolute shrinkage and selection operator and multivariate logistic regression were used to select predictors and build a nomogram. Model performance was evaluated using the area under the receiver operating characteristic curve (AUC), calibration, and decision curve analysis.

**Results:**

The overall incidence of RBP was 17.5%. The final model identified eight independent predictors. Alongside established factors like greater fracture burden and lower cement volume, reduced rCSA of the Gmax and Gmed emerged as significant and strong risk factors (*p* = 0.012 and *p* < 0.001, respectively). The nomogram demonstrated excellent discrimination in the training cohort (AUC = 0.883) and good generalizability in the validation cohort (AUC=0.695). Calibration and decision curve analysis confirmed its clinical utility.

**Conclusions:**

This study presents a practical nomogram that effectively predicts RBP risk after PVA by integrating core muscle morphology, particularly of the gluteal muscles, with conventional clinical variables. The strong association of gluteal muscle size with pain outcomes underscores the importance of a holistic muscle-bone health assessment. This tool aids in preoperative risk stratification, potentially guiding targeted prehabilitation to improve patient outcomes.

## 1 Introduction

Osteoporotic vertebral compression fractures (OVCF) represent a major public health concern for the aging population globally. As a hallmark complication of osteoporosis-a systemic skeletal disease characterized by compromised bone strength that predisposes individuals to fracture-OVCF impose a substantial burden of pain, disability, reduced quality of life, and significant healthcare costs (1, 2). For painful OVCF refractory to conservative treatment, percutaneous vertebral augmentation (PVA), including vertebroplasty (PVP) and kyphoplasty (PKP), has revolutionized management (3). These minimally invasive techniques stabilize the fractured vertebra through cement injection, providing rapid pain relief and restoring vertebral height (4). However, a significant subset of patients experiences residual back pain (RBP) postoperatively, which remains a challenging clinical dilemma (5).

RBP after PVA stems from a complex, multifactorial pathophysiological process beyond simple fracture stabilization. Patient-specific factors are crucial. Low bone mineral density (BMD), indicative of severe osteoporosis, not only predisposes to fracture but may also impair bone-cement integration and the stability of the augmented segment (6). Beyond bone quality, psychological factors such as depression significantly modify pain perception and recovery. Preoperative depressive symptoms can heighten pain sensitivity and impair coping mechanisms, contributing to persistent pain even after a technically successful procedure (5). Lifestyle factors like smoking further compromise bone health and healing (7). Fracture characteristics, including a higher vertebral fracture burden and the presence of an intravertebral vacuum cleft (often linked to Kummell's disease), signify greater spinal fragility and may correlate with persistent pain (3). The quality of cement augmentation is paramount. Insufficient volume or maldistribution can lead to inadequate stabilization, while cement leakage may cause direct neural compression or thermal injury. Furthermore, long-term failure at the cement-bone interface can result in mechanical recurrence of pain (8).

Emerging evidence highlights the importance of the paravertebral muscular system in spinal health and pain generation. The paraspinal muscles (e.g., multifidus, erector spinae) are crucial for dynamic spinal stability. Sarcopenia, the loss of muscle mass and strength, is associated with poor outcomes in various spinal disorders (9). Specifically, reduced paraspinal muscle area and density correlate with lumbar spine degeneration, including disc degeneration and facet joint arthritis (10). This relationship suggests that OVCF patients with concomitant paraspinal sarcopenia may have impaired dynamic stabilization. This places greater stress on the augmented vertebra and adjacent segments, potentially manifesting as RBP. Similarly, the gluteal muscles (gluteus maximus and medius) play an integral role in pelvic stability and gait mechanics. Weakness or dysfunction in these muscles can alter lumbopelvic kinematics, potentially contributing to persistent low back pain even after vertebral fracture stabilization (11). The thoracolumbar fascia, a complex connective tissue structure, is another element in this myofascial pain network. Injury or chronic strain to the fascia, which may occur concurrently with or be exacerbated by vertebral collapse, can be a direct source of nociceptive input and is often overlooked in evaluating post-PVA pain (12).

Given the multifactorial nature of RBP following PVA, accurately identifying high-risk patients preoperatively remains a substantial clinical challenge. Moving beyond the traditional bone-centric focus on BMD and cement augmentation, we propose that a comprehensive assessment of musculoskeletal integrity, particularly of the stabilizing core muscles, is crucial. To address this, we aimed to develop and validate a practical predictive nomogram that explicitly integrates the morphology of the paravertebral and pelvic musculature with conventional demographic, radiographic, and procedural variables. This novel synthesis aims to enable personalized risk stratification by accounting for the patient's global musculoskeletal capacity rather than vertebral stability alone. Ultimately, this paradigm shift toward a holistic muscle-bone assessment holds promise for refining patient selection, guiding tailored prehabilitation, and improving outcomes in OVCF management.

## 2 Materials and methods

### 2.1 Patient data

This study was approved by the Ethics Committees of Zhongnan Hospital of Wuhan University and The First Affiliated Hospital of Henan University of Chinese Medicine. Clinical data from patients with OVCF admitted to the two participating centers between January 2020 and December 2023 were retrospectively collected. The diagnosis of OVCF was based on the principal discharge diagnosis recorded in the hospital's medical record system, corresponding to ICD-10 code M80 (osteoporosis with pathological fracture).

### 2.2 Inclusion and exclusion criteria

Patients diagnosed with OVCF who underwent PVA were considered for this study. The inclusion criteria were as follows: (1) age ≥50 years; (2) a primary diagnosis of acute or subacute OVCF confirmed by clinical symptoms and magnetic resonance imaging; (3) performance of PVA (vertebroplasty or kyphoplasty) as the primary surgical intervention; and (4) availability of complete preoperative, intraoperative, and postoperative follow-up data, including assessments of back pain.

Patients were excluded based on the following criteria: (1) vertebral fractures caused by high-energy trauma or underlying malignancy; (2) presence of severe neurological deficits requiring decompressive surgery; (3) history of previous spinal surgery at the fractured level; (4) concomitant active infection or systemic inflammatory disease; (5) inability to complete the required follow-up evaluations or questionnaires due to cognitive impairment or other severe comorbidities; and (6) loss to follow-up before the primary outcome assessment at the designated time point.

### 2.3 Percutaneous vertebral augmentation procedure

All PVA procedures were performed by specialized spinal surgery teams following standardized institutional protocols. Under continuous fluoroscopic guidance, a working cannula was percutaneously advanced into the anterior third of the affected vertebral body. The surgical approach (unilateral or bilateral transpedicular) and the decision to use balloon kyphoplasty were based on anatomical assessment and the degree of vertebral height loss, respectively. Polymethylmethacrylate bone cement was prepared at a standard powder-to-liquid ratio of 3:1 and injected during its high-viscosity “toothpaste” phase. The volume was titrated by vertebral level, targeting 2–6 mL for thoracic and 4–8 mL for lumbar vertebrae, with the goal of achieving stabilization while preventing extravasation. Injection ceased upon satisfactory vertebral filling or the first sign of paravertebral leakage.

### 2.4 Data collection and definitions

We recorded demographic and clinical parameters including age, sex, smoking history, diabetes, depression status, and glucocorticoid use. BMD of lumbar spine (L1-L4) was measured by dual-energy x-ray absorptiometry, with osteoporosis defined as a T-score ≤2.5. Preoperative magnetic resonance imaging identified fracture levels and assessed thoracolumbar fascia injury (TLFI). Preoperative computed tomography evaluated the presence of an intravertebral vacuum cleft. Cement volume data came from surgical records, and maldistribution was assessed on postoperative radiographs. Pain intensity was measured using the visual analogue scale (VAS), a widely adopted patient-reported outcome measure for low back pain (13, 14). Preoperative and 1-month postoperative VAS scores were recorded. For this study, RBP was defined as a VAS score ≥4 at the 1-month follow-up.

### 2.5 Pelvic and paravertebral musculature quantification

Truncal computed tomography images were analyzed using the Muscle + Fat mode in Carestream software (version 12.1) for tissue segmentation.

**Figure 1:**
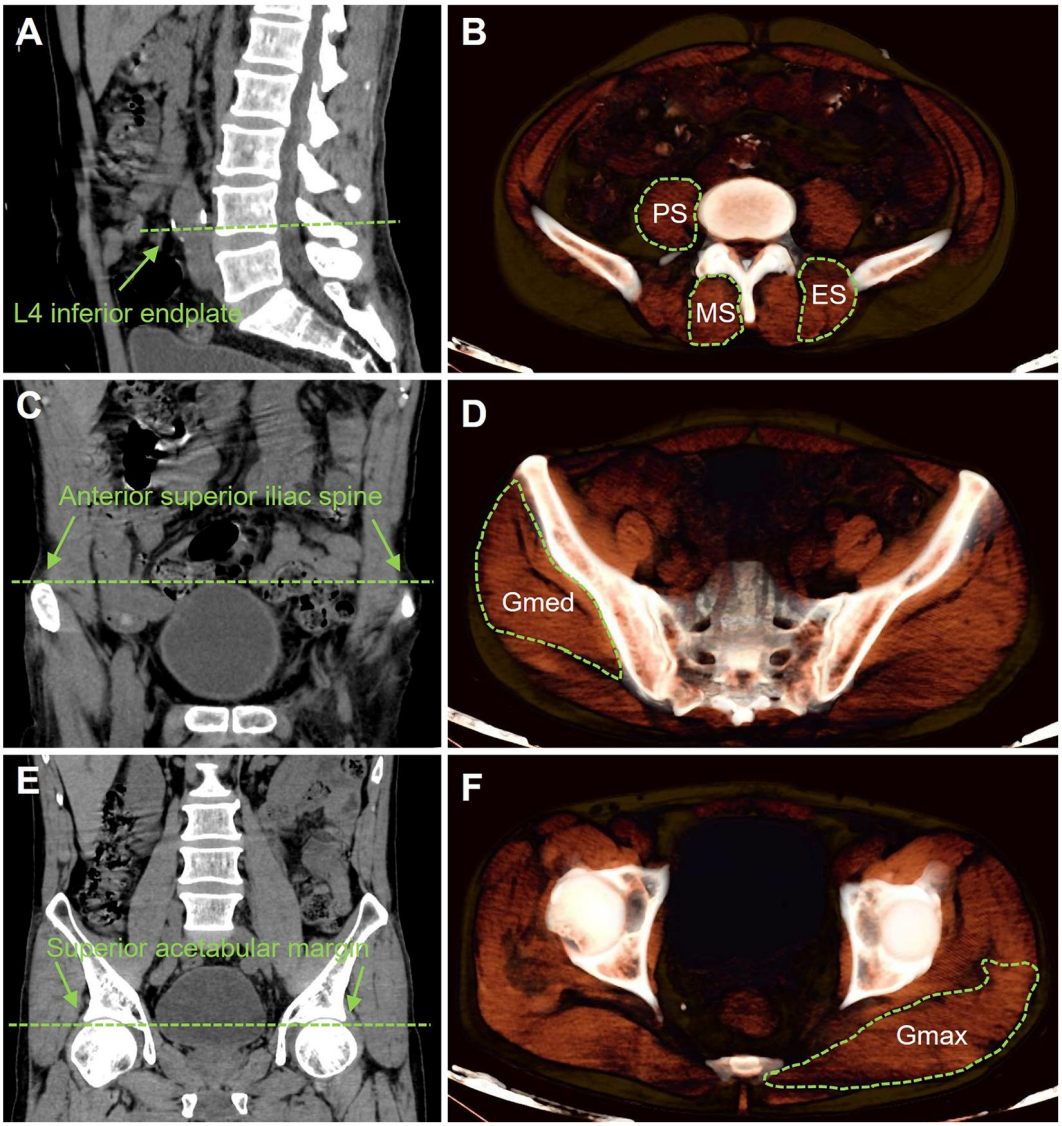
Measurement of cross-sectional area for core muscle morphology. **(A)** Sagittal CT image at the level of the inferior endplate of L4. **(B)** Corresponding axial cross-sectional area for psoas major, multifidus, and erector spinae muscles. **(C)** Coronal CT image at anterior superior iliac spine level. **(D)** Corresponding axial cross-sectional area for Gluteus medius muscles. **(E)** Coronal CT image at upper-acetabular margin level. **(F)** Corresponding axial cross-sectional area for gluteus maximus muscles.

The cross-sectional area (CSA) of the multifidus (MF), erector spinae (ES), and psoas major (PS) muscles was measured bilaterally at the L4 lower endplate level (Figures 1A,B). The CSA of the gluteus medius (Gmed) and gluteus maximus (Gmax) was measured at the anterior superior iliac spine and the superior acetabular margin, respectively (Figures 1C,F). These anatomical landmarks were selected following the standardized protocol by Lee et al., ensuring consistency and comparability with contemporary muscle morphology assessment studies (11). To normalize for body habitus, the L4 vertebral body CSA was also measured. The relative CSA (rCSA) for each muscle was calculated as (muscle CSA/L4 vertebral body CSA).

All measurements were performed by a single experienced spine surgeon who was completely blinded to patient demographic characteristics, clinical presentations, and postoperative outcomes. Blinding was maintained throughout the measurement process to minimize potential measurement bias and ensure objective quantification. The surgeon possessed extensive experience in CT-based muscle morphology assessment, having performed similar measurements in over 600 previous cases. The same blinded surgeon performed a second independent measurement on 50 randomly selected cases at least 2 weeks after the initial measurements, with complete memory washout of the first measurements. Intraclass correlation coefficient (ICC) = 0.85 (95% CI: 0.78–0.91) for all muscle measurements, indicating excellent reliability and reproducibility.

### 2.6 Statistical analysis

Continuous variables are presented as mean ± standard deviation, and compared using Student's *t*-test or the Mann–Whitney *U* test, as appropriate. Categorical variables were analyzed using the chi-square or Fisher's exact test. In the training cohort, the least absolute shrinkage and selection operator (LASSO) logistic regression was used to select independent predictors and build the RBP prediction nomogram. Model performance was evaluated using the receiver operating characteristic (ROC) curve, calibration curve, and decision curve analysis (DCA). In our DCA curve, the model provides net benefit across a wide range of threshold probabilities from 0% to 70%. The area under the ROC curve (AUC) was reported. A *p*-value < 0.05 was considered statistically significant. Analyses were performed using IBM SPSS Statistics (version 23.0) and R (version 4.2.2).

## 3 Results

### 3.1 Patient characteristics

The baseline demographic and clinical characteristics of the 428 patients with 75 RBP (17.5%) included in this predictive study are summarized. The dataset was randomly split into training and validation cohorts in a 3:1 ratio, the training cohort comprised 321 patients with 60 RBP (18.7%), while the internal test cohort included 107 patients with 15 RBP (14.0%). The two cohorts were well-balanced across most baseline variables (Table 1). Specifically, there were no statistically significant differences (*p* > 0.05) in age, sex distribution, smoking status, prevalence of diabetes or depression, glucocorticoid use, BMD of lumbar spine, number of treated vertebral segments, preoperative VAS score, presence of intravertebral vacuum cleft or TLFI, choice of surgical procedure (PVP vs. PKP), injected cement volume, incidence of cement maldistribution, or 1-month postoperative VAS score. Muscle architecture parameters, including the rCSA of the Gmed, PS, ES, and MF, also showed no significant inter-cohort differences (Table 1). The only variable exhibiting a statistically significant difference was the rCSA of the Gmax, which was slightly higher in the test cohort (6.31 ± 0.75 vs. 6.13 ± 0.88, *p* = 0.046).

**Table 1:** Patient demographics and baseline characteristics.

Characteristic	Cohort		*p*-value
	Training cohort *N* = 321	Internal Test Cohort *N* = 107	
Age, Mean ± SD	67 ± 11	69 ± 12	0.212^1^
Sex, *n* (%)			0.134^2^
Male	52 (16.2%)	11 (10.3%)	
Female	269 (83.8%)	96 (89.2%)	
Smoking, *n* (%)			0.217^2^
No	259 (80.7%)	92 (86.0%)	
Yes	62 (19.3%)	15 (14.0%)	
Diabetes, *n* (%)			0.345^2^
No	247 (76.9%)	87 (81.3%)	
Yes	74 (23.1%)	20 (18.7%)	
Depression, *n* (%)			0.723^2^
No	284 (88.5%)	96 (89.7%)	
Yes	37 (11.5%)	11 (10.3%)	
Glucocorticoid, *n* (%)			0.590^2^
No	299 (93.1%)	98 (91.6%)	
Yes	22 (6.9%)	9 (8.4%)	
BMD of LL, Mean ± SD	−3.23 ± 1.20	−3.11 ± 1.10	0.363^1^
Segment, Mean ± SD	1.18 ± 0.50	1.19 ± 0.52	0.973^1^
Pre-VAS, Mean ± SD	4.96 ± 1.37	4.79 ± 1.34	0.257^1^
TLFI, *n* (%)			0.760^2^
No	271 (84.4%)	89 (83.2%)	
Yes	50 (15.6%)	18 (16.8%)	
Vacuum cleft, *n* (%)			0.789^2^
No	285 (88.8%)	96 (89.7%)	
Yes	36 (11.2%)	11 (10.3%)	
Treatment, *n* (%)			0.198^2^
PVP	118 (36.8%)	32 (29.9%)	
PKP	203 (63.2%)	75 (70.1%)	
Cement volume, Mean ± SD	5.66 ± 1.05	5.57 ± 0.98	0.414^1^
Maldistribution, *n* (%)			0.576^2^
No	277 (86.3%)	90 (84.1%)	
Yes	44 (13.7%)	17 (15.9%)	
rCSA of Gmax, Mean ± SD	6.13 ± 0.88	6.31 ± 0.75	0.046^1^
rCSA of Gmed, Mean ± SD	4.07 ± 0.79	4.07 ± 0.62	0.945^1^
rCSA of PS, Mean ± SD	1.29 ± 0.21	1.32 ± 0.26	0.218^1^
rCSA of ES, Mean ± SD	1.57 ± 0.26	1.60 ± 0.26	0.329^1^
rCSA of MF, Mean ± SD	0.87 ± 0.23	0.93 ± 0.27	0.070^1^
1 month-VAS, Mean ± SD	1.99 ± 1.31	1.86 ± 1.22	0.357^1^

^1^ Welch two sample *t*-test.

^2^ Pearson's chi-squared test.

### 3.2 Variables screening using LASSO regression

**Figure 2:**
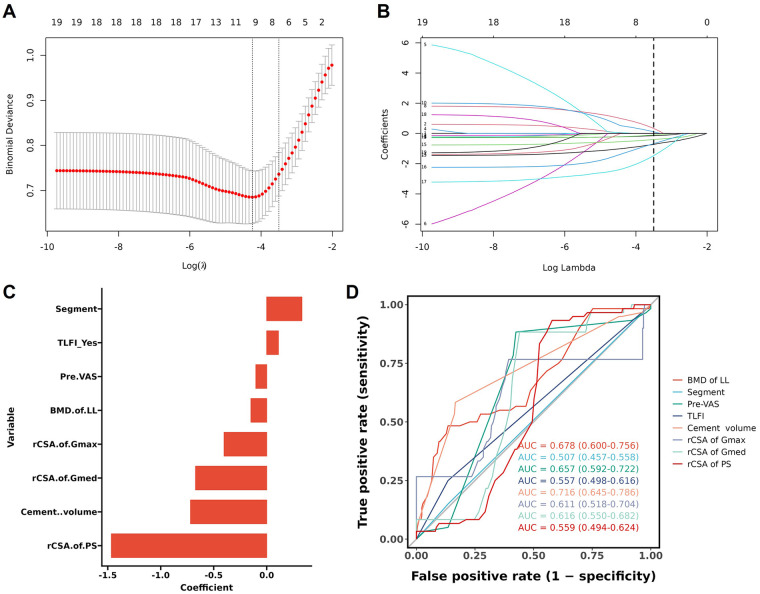
Variables screening using LASSO regression. **(A)** 10-fold cross-validation was applied to select the most suitable feature using the LASSO regression model. **(B)** Plot of the LASSO regression coefficient profiles. **(C)** Only variables with non-zero coefficients after LASSO selection are shown. **(D)** Receiver operating characteristic curves for individual predictors assessed in univariate logistic regression.

Since there were too many variables, it is difficult to reduce the influence of multicollinearity among variables, confounding factors, and other problems by conducting only univariate analysis, so this study used the LASSO regression for variables screening to obtain non-zero coefficient variables (Figures 2A,B). The candidate predictors, age, sex, smoking, diabetes, depression, glucocorticoid, BMD of lumbar spine, segment, pre-VAS, TLFI, vacuum cleft, treatment (PKP or PVP), cement volume, maldistribution, rCSA of Gmax, rCSA of Gmed, rCSA of PS, rCSA of ES, and rCSA of MF, were included in the original model, which were then reduced to 8 potential predictors using LASSO regression analysis performed in the training cohort (Figure 2C).

The receiver operating characteristic curves for individual predictors of RBP are presented, with the AUC values and their corresponding 95% confidence interval (CI) indicating the discriminatory performance of each variable. The ROC analysis of the abovementioned variables yielded AUC values greater than 0.5 (Figure 2D). Cement volume demonstrated the highest discriminatory ability (AUC = 0.716, 95% CI: 0.645–0.786), followed by BMD of lumbar spine (AUC = 0.678, 95% CI: 0.600–0.756) and pre-VAS (AUC = 0.657, 95% CI: 0.592–0.722). The variables rCSA of Gmed (AUC = 0.616, 95% CI: 0.550–0.682) and rCSA of Gmax (AUC = 0.611, 95% CI: 0.518–0.704) showed moderate discriminatory performance, while TLFI (AUC = 0.557, 95% CI: 0.498–0.616), rCSA of PS (AUC = 0.559, 95% CI: 0.494–0.624), and Segment (AUC = 0.507, 95% CI: 0.457–0.558) exhibited relatively lower discriminatory power, with the AUC for Segment approaching the null value of 0.5.

### 3.3 Multivariate analysis

Further multivariate logistic analyses were carried out in training cohorts by constructing the optimal logistic regression model using non-zero coefficient variables selected by the Lasso method. In the multivariate logistic regression analysis of the training cohort, several factors were significantly associated with the outcome of RBP. Specifically, the number of fracture segments (OR: 5.27, 95% CI: 2.20–12.64, *p* < 0.001), TLFI (OR: 2.71, 95% CI: 1.04–7.08, *p* = 0.041) were identified as significant predictors. Additionally, reduced cement volume (OR: 0.23, 95% CI: 0.14–0.38, *p* < 0.001), rCSA of the Gmax (OR: 0.49, 95% CI: 0.28–0.85, *p* = 0.012), Gmed (OR: 0.05, 95% CI: 0.01–0.25, *p* < 0.001), and PS muscles (OR: 0.04, 95% CI: 0.00–0.39, *p* = 0.005) were also significantly associated with the outcome. In contrast, BMD of lumbar spine (OR: 0.70, 95% CI: 0.48–1.03, *p* = 0.073) and preoperative VAS score (OR: 0.79, 95% CI: 0.59–1.06, *p* = 0.118) did not demonstrate statistically significant associations in this model. Results are shown in the following Table 2.

**Table 2:** Results of multivariate logistic regression for training cohort.

Characteristic	*N*	Event *N*	OR	95% CI	*p*-value
BMD of LL	321	60	0.70	0.48, 1.03	0.073
Segment	321	60	5.27	2.20, 12.64	<0.001
Pre-VAS	321	60	0.79	0.59, 1.06	0.118
TLFI					
No	269	45	—	—	
Yes	52	15	2.71	1.04, 7.08	0.041
Cement volume	321	60	0.23	0.14, 0.38	<0.001
rCSA of Gmax	321	60	0.49	0.28, 0.85	0.012
rCSA of Gmed	321	60	0.05	0.01, 0.25	<0.001
rCSA of PS	321	60	0.04	0.00, 0.39	0.005

CI, confidence interval; OR, odds ratio.

### 3.4 Establishment and validation of nomogram

**Figure 3:**
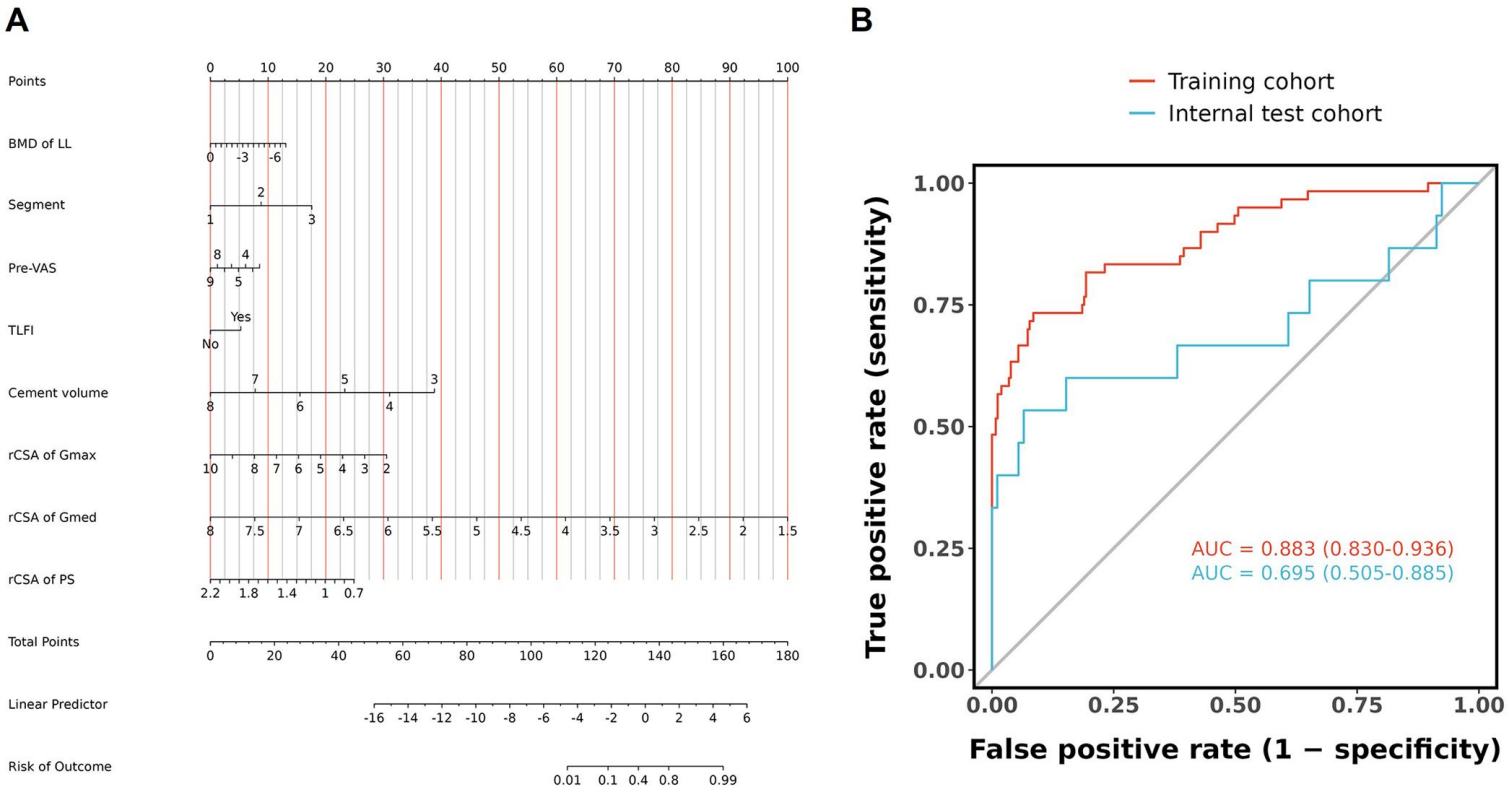
Establishment of the predictive nomogram. **(A)** Visualisation nomogram of logistic regression for residual low back pain after percutaneous vertebral augmentation. **(B)** The receiver operating characteristic curve analysis for training and validation cohorts.

The final logistic model included 8 independent predictors (BMD of lumbar spine, segment, pre-VAS, TLFI, cement volume, rCSA of Gmax, rCSA of Gmed, and rCSA of PS) and was developed as a simple-to-use nomogram, which is illustrated in the following Figure 3A. The receiver operating characteristic curve analysis demonstrated that the predictive model achieved an AUC of 0.883 (95% CI: 0.830–0.936) for the outcome of RBP in the training cohort, indicating robust discriminative performance. In the independent validation cohort, the model's performance was maintained with an AUC of 0.695 (95% CI: 0.505–0.885). The AUCs of the model in the different cohorts were shown in the following Figure 3B.

**Figure 4:**
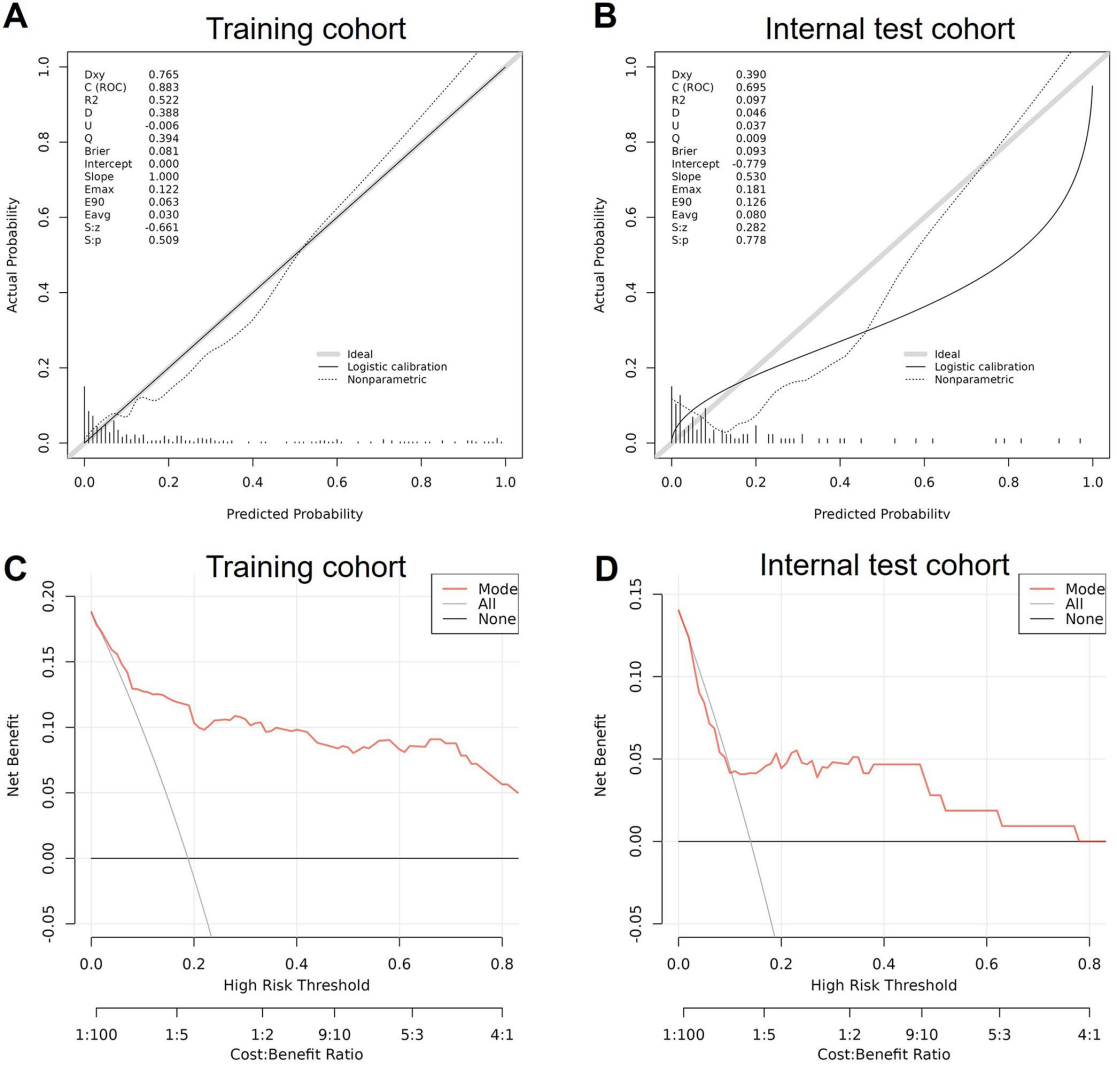
Validation of the nomogram. **(A)** The calibration plots of the nomogram in the training cohort. **(B)** The calibration plots of the nomogram in the validation cohort. **(C)** The decision curve analysis related to the nomogram in the training cohort. **(D)** The decision curve analysis related to the nomogram in the validation cohort.

The calibration plots of the nomogram in the different cohorts are plotted in Figures 4A,B, which demonstrates a good correlation between the observed and predicted RBP. The results showed that the original nomogram was still valid for use in the validation sets, and the calibration curve of this model was relatively close to the ideal curve, which indicates that the predicted results were consistent with the actual findings. The following Figures 4C,D displays the DCA curves related to the nomogram, which showed that the nomogram provided a net clinical benefit across a range of high-risk threshold probabilities.

## 4 Discussion

This study developed and validated a nomogram for predicting RBP following PVA in patients with OVCF. Notably, a key innovation of this model is its incorporation of core muscle morphology metrics, advancing the predictive framework from one focused predominantly on bone quality and surgical technique to one that embodies an integrated muscle-bone health perspective. The multivariate logistic regression analysis identified several independent predictors, which can be broadly categorized into fracture characteristics, procedural factors, and-as a novel category-muscle morphology. The presence of a segmental fracture emerged as a strong predictor, with an odds ratio exceeding five. This aligns with the established understanding that more extensive spinal injury portends a worse prognosis, as a higher number of fractured vertebrae has been consistently linked to adverse outcomes, including new fractures (9). The identification of thoracolumbar fascia injury as a significant factor further underscores the importance of soft tissue integrity beyond the bony fracture itself. Research indicates that lumbar fascial edema is associated with a higher incidence of postoperative residual pain, and factors like advanced age and trauma history contribute to such soft tissue injury (15). This finding expands the conceptual framework for understanding post-procedural pain, moving beyond vertebral stabilization to include the healing of surrounding supportive structures.

A particularly novel and significant finding of this study is the strong association between reduced relative cross-sectional area of the gluteal and psoas muscles and RBP. This highlights a previously underexplored connection between global musculoskeletal health and localized spinal outcomes. The rCSA of the gluteus medius and psoas showed remarkably high protective odds ratios, indicating that sarcopenia or regional muscle atrophy is a potent risk factor for poor pain relief after PVA. Recently, several high-quality randomized controlled trials have provided additional evidence supporting the association between core muscle morphology and spinal prognosis. Li et al. investigated the effects of active spinal orthosis on fatty infiltration in paraspinal muscles in women with osteoporotic vertebral fracture, demonstrating that orthosis intervention could attenuate muscle fatty infiltration and improve clinical outcomes (16). Güler et al. reported that core stabilization exercises significantly improved lumbar multifidus morphology and functional outcomes in chronic non-specific low back pain patients through a randomized controlled trial (17). Furthermore, Zhou et al. evaluated the effects of core stability exercises combined with Russian electrical stimulation on pain perception and muscle function, confirming the therapeutic value of core muscle rehabilitation (18). These recent findings collectively reinforce the importance of core muscle assessment in spinal disease management, aligning with our nomogram's emphasis on gluteal muscle morphology as a predictor of residual pain after vertebral augmentation.

The superior performance of muscle parameters relative to BMD may reflect several factors. While BMD measures bone quantity, muscle morphology directly reflects the neuromuscular system's capacity for dynamic stabilization of the spine. For pain outcomes after augmentation, functional stabilization capacity may be more relevant than static bone density. Muscle morphology integrates multiple aspects of systemic health (nutrition, hormonal status, physical activity level, inflammation), whereas BMD represents a single parameter. Importantly, muscle morphology is potentially modifiable through targeted exercise and rehabilitation, whereas BMD changes more slowly (19). This therapeutic actionability enhances the clinical utility of muscle-based assessment. Gluteal and psoas muscles are directly involved in lumbopelvic biomechanics and spinal load distribution. Their dysfunction directly impairs spinal stability, more directly impacting pain outcomes than bone density alone (20). Therefore, rather than muscle morphology parameters merely complementing BMD assessment, our data suggest they provide superior and more clinically actionable information for predicting RBP risk. This paradigm shift—from BMD-centric assessment to muscle-inclusive holistic evaluation—represents a meaningful advancement in the field.

Although BMD did not achieve conventional statistical significance in our multivariable model (*p* = 0.073), several methodological and clinical considerations warrant careful interpretation of this finding. Our study population consisted exclusively of patients with osteoporotic vertebral compression fractures, inherently restricting the range and variance of BMD values. This range restriction is a well-recognized statistical phenomenon that attenuates effect sizes and reduces the discriminatory power of predictors, even when true underlying associations exist (21). Within such a homogeneous cohort, the compressed BMD distribution may be insufficient to exert a strong discriminatory influence on short-term pain outcomes, despite its established pathophysiological role in vertebral bone quality, load-bearing capacity, and bone-cement integration. Indeed, BMD remains a robust predictor of subsequent fracture risk, long-term spinal stability, and the need for future interventions in broader clinical contexts, and continues to guide critical decisions regarding augmentation appropriateness and anti-osteoporotic pharmacotherapy (9, 22, 23). Therefore, the marginally non-significant result in our model should not diminish the clinical importance of BMD assessment but rather be contextualized as a consequence of cohort homogeneity and the short-term nature of our outcome measure; muscle morphology may serve as a more sensitive discriminator of acute residual back pain within this already-osteoporotic population, complementing rather than replacing BMD in comprehensive patient evaluation.

Several predictive models for RBP after vertebral augmentation have been previously reported. A radiomics-based nomogram using preoperative CT images achieved an AUC of 0.823 in the training cohort (24). Recently, a combined model integrating CT-based paraspinal muscle radiomics with clinical features was constructed to predict residual low back pain after percutaneous kyphoplasty, demonstrating good predictive performance (25). Machine learning-based models and CT radiomics prediction models have also been explored for RBP prediction after PVA (26, 27). However, these existing models primarily focused on bone-related parameters, radiographic features, or radiomics signatures, without incorporating direct measurements of core muscle morphology, particularly pelvic muscles such as the gluteus maximus and gluteus medius. Our study is the first to integrate comprehensive core muscle morphology—including both paravertebral muscles and pelvic muscles—into a clinical nomogram for RBP prediction after PVA. The inclusion of gluteal muscle parameters represents a novel addition that may capture the functional relationship between trunk stability and postoperative pain outcomes.

The discriminative performance of the model was robust, with an AUC of 0.883 in the training cohort. The maintained AUC of 0.695 in the independent validation cohort, while indicating good generalizability, also reflects the expected attenuation in performance when applying a model to a new population. This performance is comparable to other advanced prediction tools in the field. For instance, a combined clinical and radiomics model for predicting residual low back pain after kyphoplasty reported AUCs of 0.905 and 0.891 in training and testing cohorts, respectively (25). Another nomogram focusing on re-fracture risk achieved an AUC of 0.886 (28). Our model's strength lies in its integration of easily obtainable clinical, radiographic, and procedural variables into a practical nomogram, without requiring complex radiomic analyses, enhancing its potential for clinical adoption. Clinically, this nomogram provides a practical tool for spine specialists to perform individualized risk stratification during preoperative counseling. Patients with high-risk scores, characterized by segmental fractures, TLFI, low cement volume, and poor muscle morphology, can be identified for closer monitoring and more aggressive multimodal management. This may include structured prehabilitation or postoperative rehabilitation programs focused on core strengthening, given the identified importance of gluteal and psoas muscles.

This study has several limitations. First, as a retrospective study from a single center, potential selection bias cannot be completely eliminated. Second, several factors known to influence RBP were not included in our analysis, such as fracture chronicity/acuteness, bone marrow edema status, osteoporosis treatment history, and postoperative rehabilitation interventions. Although we adjusted for multiple established clinical predictors, residual confounding cannot be fully excluded. For example, patients with acute fractures may have different pain trajectories compared to chronic cases, and those receiving aggressive osteoporosis treatment or postoperative rehabilitation may demonstrate better pain outcomes. The absence of these variables may lead to overestimation or underestimation of the true effect of muscle morphology on RBP. Third, RBP was defined solely as VAS score ≥4 at 1 month postoperatively without distinguishing between acute residual pain and chronic pain. Additionally, long-term follow-up data at 3 months and 6 months were not available, limiting the assessment of the model's long-term prognostic value. Fourth, the relatively small sample size in the validation cohort may limit the generalizability of our findings. Fifth, muscle morphology was assessed only at baseline, and longitudinal changes during follow-up were not analyzed. Future prospective studies with larger sample sizes, multi-center validation, comprehensive confounder adjustment, and extended follow-up are warranted to confirm our findings.

In conclusion, this study successfully developed a predictive nomogram for RBP after PVA, highlighting the multifactorial nature of this outcome. The model identifies key risk factors spanning fracture severity, procedural details, and systemic muscle health, moving beyond a narrow focus on bone density alone. The strong protective association of greater cement volume and larger core muscle cross-sectional area offers actionable targets for surgical technique and preoperative optimization. By translating these findings into a clinically usable tool, this work facilitates personalized risk assessment and supports a more comprehensive, integrated management strategy for patients with OVCF, ultimately aiming to improve functional recovery and quality of life.

## Data Availability

The raw data supporting the conclusions of this article will be made available by the authors, without undue reservation.

## References

[1] Schloemann DT Lane CY Rubery PT Thirukumaran CP. Sociodemographic differences in the epidemiology and management of osteoporosis-related vertebral fractures. Curr Osteoporos Rep. (2025) 24(1):1. 10.1007/s11914-025-00944-z 41402603

[2] Lin YH Lin J Xu JY Lai BX He MH Zhu YR What risk factors are associated with recurrent osteoporotic vertebral compression fractures after percutaneous vertebral augmentation? A meta-analysis. Clin Orthop Relat Res. (2025) 483(8):1528–39. 10.1097/CORR.0000000000003430 40036060 PMC12266891

[3] Barcena AJR Mishra A Lopez S Martin B Bolinas DKM Huang SY Novel bone materials and adjunctive approaches in percutaneous vertebral augmentation for neoplastic vertebral compression fractures. Radiology. (2025) 317(3):e243744. 10.1148/radiol.243744 41432556 PMC12728518

[4] Sun N Chu Y Ge Z Liu Y. Percutaneous vertebral augmentation for osteoporotic vertebral compression fractures: minimally invasive techniques and clinical outcomes. Eur J Med Res. (2025) 30(1):1037. 10.1186/s40001-025-03311-x 41163108 PMC12570528

[5] Lyu FF Zhang M Deng YF Liu Q Yang Q Xia LR. Incidence and predictors of residual back pain after percutaneous vertebral augmentation in osteoporotic vertebral compression fracture: a systematic review and meta analysis. Osteoporos Int. (2025) 36(10):1781–94. 10.1007/s00198-025-07609-8 40699245

[6] Wang H Zhang H Xiao C Zhang K Qi L. Risk factors of residual back pain after vertebral augmentation in osteoporotic vertebral compression fracture patients: a systematic review and meta-analysis. BMC Musculoskelet Disord. (2025) 26(1):702. 10.1186/s12891-025-08945-w 40713499 PMC12291499

[7] Yang XG Dong YQ Liu X Liu XL Luo HT Bao Y Incidence and prognostic factors of residual back pain in patients treated for osteoporotic vertebral compression fractures: a systematic review and meta-analysis. Eur Spine J. (2024) 33(12):4521–37. 10.1007/s00586-024-08426-z 39103616

[8] Daher M Aoun M Xu A Daniels AH Sebaaly A. Is there a difference in postoperative outcomes between kyphoplasty and vertebroplasty in the management of vertebral compression fractures?: a meta-analysis of randomized controlled trials. J Bone Joint Surg Am. (2025) 107(17):1967–74. 10.2106/JBJS.24.01191 40690559

[9] Liu S Sun Y Man Y He M Huang P. Risk factors for new vertebral compression fracture following percutaneous vertebral augmentation: a systematic review and meta-analysis based on multivariate logistic regression analysis. J Orthop Surg Res. (2025) 20(1):1016. 10.1186/s13018-025-06424-5 41257820 PMC12628626

[10] Weng Y Wang L Huang J Cai L. Factors associated with new fractures in adjacent vertebrae after percutaneous vertebroplasty for osteoporotic vertebral compression fractures. Am J Transl Res. (2024) 16(11):6972–9. 10.62347/WBBN6996 39678623 PMC11645567

[11] Lee JM Lee DH Chung NS Chung HW Koh JH Yoon Y Hip, abdomen, and paraspinal muscle morphologies and their correlation with pain and disability in degenerative lumbar scoliosis patients. Spine. (2025) 50(22):1589–96. 10.1097/BRS.0000000000005362 40237206

[12] Ahmed Mohamed A Xuyang X Zhiqiang Z Chen J. Association between thoracolumbar fascia injury and residual back pain following percutaneous vertebral augmentation: a systematic review and meta-analysis. Front Endocrinol (Lausanne). (2025) 16:1532355. 10.3389/fendo.2025.1532355 40331146 PMC12052568

[13] Huskisson EC. Measurement of pain. Lancet (London, England). (1974) 2(7889):1127–31. 10.1016/S0140-6736(74)90884-8 4139420

[14] Scott J Huskisson EC. Graphic representation of pain. Pain. (1976) 2(2):175–84. 10.1016/0304-3959(76)90113-5 1026900

[15] Li N Lu Y Zhang Y. Risk factors for lumbar fascial edema in patients with osteoporotic vertebral compression fractures and its effect on residual pain after percutaneous vertebroplasty. Am J Transl Res. (2025) 17(9):6931–40. 10.62347/IMRE7659 41113003 PMC12531560

[16] Hiller M Kohl M Chaudry O Engelke K von Stengel S Kemmler W. Effects of active spinal orthosis on fatty infiltration in paraspinal muscles in kyphotic women with osteoporotic vertebral fracture-sub-analysis of a randomized controlled trial. Healthcare (Basel). (2025) 13(11):1262. 10.3390/healthcare13111262 40508875 PMC12154191

[17] Güler MA Demirdel E Gezer İA Nayman A Tezcan EA. Effect of core stabilization exercises on lumbar multifidus morphology and functional outcomes in chronic non-specific low back pain: a randomized controlled trial. BMC Musculoskelet Disord. (2025) 27(1):83. 10.1186/s12891-025-09433-x 41455992 PMC12860132

[18] Nayel N Ezzat H Ahmed S Saleh H. Effect of core stability exercises and Russian electrical stimulation on nonspecific low back pain: a single-blinded randomized controlled trial. Sci Rep. (2025) 15(1):44053. 10.1038/s41598-025-28313-x 41407791 PMC12715220

[19] Rabieezadeh A Mahdavinejad R Sedehi M Adimi M. The effects of an 8-week dynamic neuromuscular stabilization exercise on pain, functional disability, and quality of life in individuals with non-specific chronic low back pain: a randomized clinical trial with a two-month follow-up study. BMC Sports Sci Med Rehabil. (2024) 16(1):161. 10.1186/s13102-024-00948-9 39054527 PMC11271024

[20] Mallio CA Russo F Vadalà G Papalia R Pileri M Mancuso V The importance of psoas muscle on low back pain: a single-center study on lumbar spine MRI. N Am Spine Soc J. (2024) 18:100326. 10.1016/j.xnsj.2024.100326 38947493 PMC11214412

[21] Sackett PR. Yang H. Correction for range restriction: an expanded typology. J Appl Psychol. (2000) 85(1):112–8. 10.1037/0021-9010.85.1.112 10740961

[22] Shen L Yang H Zhou F Jiang T Jiang Z. Risk factors of short-term residual low back pain after PKP for the first thoracolumbar osteoporotic vertebral compression fracture. J Orthop Surg Res. (2024) 19(1):792. 10.1186/s13018-024-05295-6 39587591 PMC11590304

[23] Wang ZW Wang GY Liu DK Zhang DZ Zhao C. Risk factors for residual back pain after PVP treatment for osteoporotic thoracolumbar compression fractures: a retrospective cohort study. World Neurosurg. (2023) 180:e484–93. 10.1016/j.wneu.2023.09.094 37774786

[24] Ge C Chen Z Lin Y Zheng Y Cao P Chen X. Preoperative prediction of residual back pain after vertebral augmentation for osteoporotic vertebral compression fractures: initial application of a radiomics score based nomogram. Front Endocrinol (Lausanne). (2022) 13:1093508. 10.3389/fendo.2022.1093508 36619583 PMC9816386

[25] Jiang JL Yu R Zhang Y Ge T Xiao B He D Developing and validating a combined model with CT-based paraspinal muscle radiomics and clinical features to predict residual low back pain after percutaneous kyphoplasty. Eur Spine J. (2026) 35(3):1172–82. 10.1007/s00586-025-09627-w 41307715

[26] Wu H Li C Song J Zhou J. Developing predictive models for residual back pain after percutaneous vertebral augmentation treatment for osteoporotic thoracolumbar compression fractures based on machine learning technique. J Orthop Surg Res. (2024) 19(1):803. 10.1186/s13018-024-05271-0 39609923 PMC11603673

[27] Zhang YF Meng X. A clinical and CT-based radiomics prediction model study on residual back pain after vertebral augmentation for osteoporotic vertebral compression fractures. Asian J Surg. (2024):S1015-9584(24)01496-9. 10.1016/j.asjsur.2024.07.125 39019758

[28] Qi B Wu Q Chen G Zhang L Meng C Wei W Predicting re-fracture risk factors in older adult osteoporotic vertebral fractures patients with comorbidities: development and validation of nomogram. Front Med (Lausanne). (2025) 12:1664157. 10.3389/fmed.2025.1664157 41210860 PMC12592060

